# Developing evidence-informed pathways for long-term engagement in cycling for the United States

**DOI:** 10.3389/fspor.2026.1770224

**Published:** 2026-06-11

**Authors:** Alexandra K. Tyler, Michael Norton, Bradley Thordarson, Kristen Dieffenbach, Sean M. Wilson

**Affiliations:** 1 Lawrence D Longo MD, Center for Perinatal Biology, Loma Linda University School of Medicine, Loma Linda, CA, United States; 2 MSN Coaching, Watchung, NJ, United States; 3 Pedal Pedal Pedal, Los Angeles, CA, United States; 4 Center for Applied Coaching and Sport Sciences, College of Applied Human Science, West Virgina University, Morgantown, WV, United States

**Keywords:** cycling development, cycling infrastructure, long-Term athlete development (LTAD), sport engagement, youth cyclists, Youth Physical Development (YPD)

## Abstract

Cycling participation in the United States remains lower than in comparable nations despite widespread bicycle ownership. This manuscript examines the fragmented structure of cycling in the U.S. and proposes the adoption of evidence-informed Long-Term Athlete Development (LTAD) pathways to transform cycling from a niche pursuit into an integrated cultural practice. Drawing on structured expert discussions among coaches, program leaders, and stakeholders, this perspectives paper synthesizes practice-based insights to inform the development of a scalable framework. Although youth-oriented organizations such as USA BMX and the National Interscholastic Cycling Association demonstrate potential for scalable growth, most communities lack coherent developmental pathways linking novice riders with sustained participation. We advocate the adaptation of proven LTAD and Youth Physical Development (YPD) frameworks to establish comprehensive models spanning early childhood through lifelong engagement. Key implementation components include community-based cycling clubs functioning as local hubs, coordinated coaching education systems, and accessible programming aligned with “right age, right stage” principles. Implementation requires integrated infrastructure linking local, regional, and national organizations while prioritizing safety, affordability, and inclusion. Implementing LTAD/YPD-centered cycling networks is expected to expand participation and cultivate riders who integrate cycling into transportation, recreation, and competition. By emphasizing long-term engagement over early specialization and embedding pathways within an ecosystem of activities, relationships, and settings, this conceptual and operational framework outlines a scalable approach to positioning cycling as an inclusive and sustainable element of American culture that supports lifelong health, well-being, and social connection.

## 1 Introduction

The focus of this perspectives manuscript is to provide a conceptually grounded, evidence-informed call to action for the development of clear, scalable, and practically oriented pathways in the sport of cycling. Rather than presenting results from a formal empirical study, this paper synthesizes practice-based insights and consensus themes derived from a series of facilitated, structured discussions among experienced cycling coaches, program directors, and organizational leaders from across the United States and internationally. The primary aim is to support long-term engagement by outlining developmental pathways that build confidence, competence, and enjoyment in cycling, ultimately fostering sustained participation across recreational, transportation, and competitive contexts. These expert-informed discussions served as the foundation for identifying common developmental priorities, structural gaps, and opportunities for growth within the current U.S. cycling ecosystem. The work is born from a series of facilitated web-based conversations among cycling directors and coaches from the United States and other countries. The primary components of the conversation frameworks and resulting conceptual framework are detailed in Table 1.

**Table 1:** Stage-specific programming within an LTAD/YPD-informed cycling framework integrating activities, relationships, and settings.

ADM group/label	Approx. ages/level	Program design elements (activities, relationships, settings)
ADM 1–2 “Love to Ride”	Ages 4–12	Overarching principles for fun and safety; alignment with target developmental needs; simple bike choices; equitable partnerships with schools and parks as low-barrier venues (settings); play-based activities and short-duration sessions (activities); foundational coaching supported by basic toolkits and positive peer interactions (relationships); minimal costs; connections to scalable learn-to-ride and youth recreation programs; and progression toward confident, joyful riders.
ADM 2–3 “Building for the Future”	Ages 13–18/collegiate	Principles balancing development and enjoyment aligned with adolescent physical, social, and cognitive needs; opportunities for multi-discipline exploration and skill-building (activities); mentorship, coaching guidance, and peer group development (relationships); club-, school-, and community-based environments with local and NGB partnerships (settings); structured but flexible training approaches and goal-setting toolkits; sustainable cost considerations; and progression toward committed, multi-discipline riders.
ADM 4a “Participation for Success”	Ages 15–18/U23 collegiate (participation-focused)	Principles prioritizing inclusion, retention, and long-term engagement; participation-oriented riding formats, group rides, and event-based experiences (activities); coaching focused on culture-building, psychological safety, and social connection (relationships); community-based clubs, parks and recreation systems, and collegiate environments (settings); moderate, health-oriented training compatible with academic and work demands; accessible cost structures; and progression toward riders who integrate cycling into their lifestyle.
ADM 4b “Performance Programs”	Ages 15–18/U23 collegiate (performance-focused)	High-performance principles within athlete-centered LTAD; structured training, competition, and performance development systems (activities); advanced coaching, mentorship, and sport science support (relationships); performance clubs, regional centers, and discipline-specific training and competition venues (settings); individualized programming, monitoring, and load management; funding structures to support travel and equipment; and progression toward athletes prepared for national and international competition.
ADM 5 “Active for life”	18–60+ - lifelong engagement	Not explicitly addressed in working group conversations; There is implied linkage to ADM 3 and 4; Lifelong engagement in cycling through recreational, fitness, and transportation-based participation (activities); sustained social connections and community engagement (relationships); accessible community environments including clubs, informal groups, and public infrastructure (settings); flexible participation pathways supporting health, well-being, and continued involvement across the lifespan.
Cross-cutting conversation dimensions (all ADM)	All ages/stages	Overarching principles structured around developmentally appropriate activities; clearly defined relationships, including coaching roles, mentorship, and peer support; and accessible, scalable settings incorporating schools, clubs, and community partnerships; alongside cost considerations, progression pathways, and system alignment to support competent, confident, and connected riders.

ADM, American Development Model.

**Figure 1:**
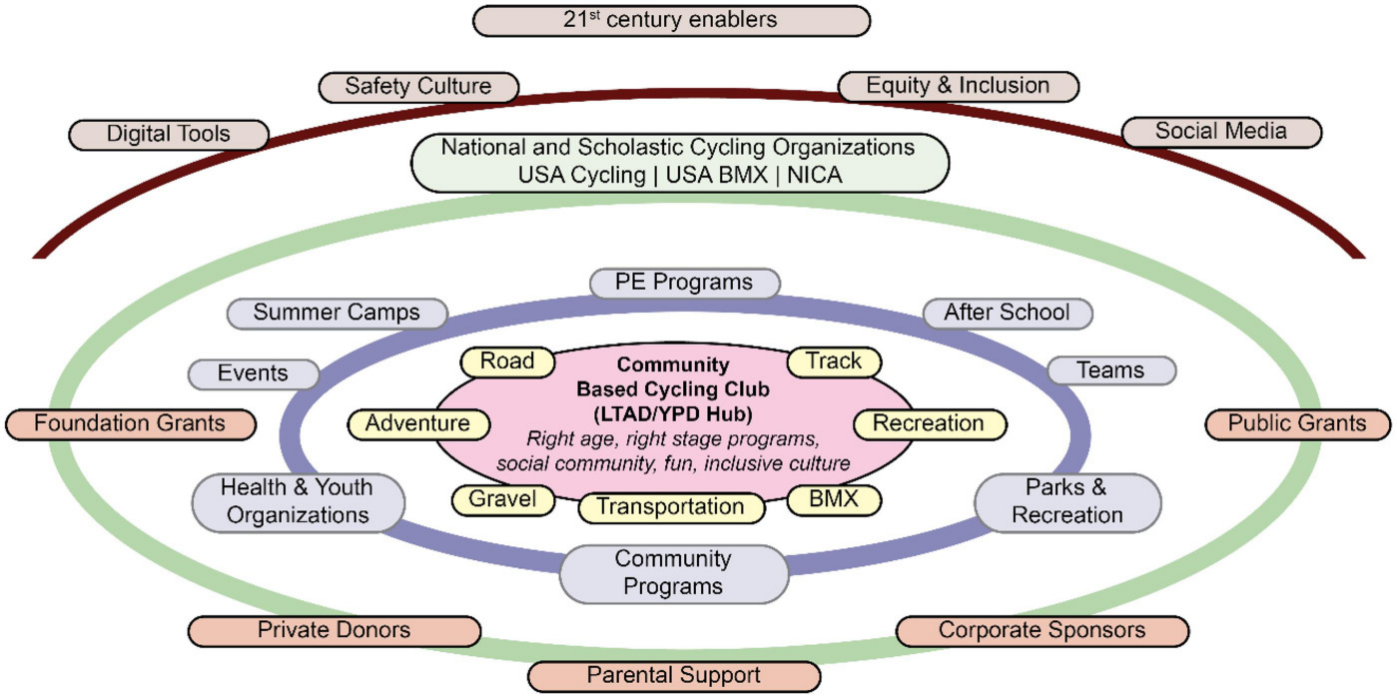
Socio-ecological development ecosystem for U.S. cycling integrating LTAD/YPD hubs and Personal Assets Framework principles.The central node represents community-based cycling clubs that function as LTAD/YPD hubs, and deliver right age, right stage programs across various disciplines (road, track, BMX, gravel, adventure, etc.). These programs can also support transportation and recreational cycling within a fun, inclusive social community. The ring and nodes surrounding the hub depict activities and partners including school-based PE programs, community programs, parks and recreation, teams, competitive and noncompetitive events, summer camps, after-school initiatives, and health and youth-serving organizations. Within this ring, coaches and coach developers are central to ensuring that youth encounter safe, developmentally appropriate experiences wherever they enter or move through the ecosystem. These coaches are supported by coherent, evidence-informed education systems. Each of these elements interconnects young riders to the hub and reinforce long-term engagement. The outer ring and nodes indicate national and scholastic cycling organizations (USA Cycling, USA BMX, NICA) and various funding sources (corporate sponsors, public and foundation grants, private donors, parental support). These provide structural, financial, and competitive scaffolding across multiple levels of the ecosystem. The upper band highlights 21st-century enablers that cut across all levels and shape how cycling pathways are discovered, experienced, and sustained in the modern US context. This includes the use of digital tools and social media, along with a strong safety culture and commitments to equity and inclusion. Viewed through the lens of the Personal Assets Framework, this model demonstrates how activities, relationships, and settings interact across multiple ecological levels to influence youth development in cycling. The figure emphasizes that long-term participation and performance outcomes are not solely determined by progression through competitive pathways, but rather by the quality, accessibility, and alignment of experiences across this interconnected system. This ecosystem-based approach provides a scalable and adaptable model for fostering lifelong engagement in cycling within the decentralized U.S. sporting landscape.

Across all ADM levels, Table 1 illustrates how developmentally appropriate activities, supportive relationships, and accessible settings interact to support long-term engagement, consistent with the Personal Assets Framework and the ecosystem model presented in Figure 1.

Cycling continues to be a world-leading activity for sport, leisure, and human-powered transportation. In the United States, cycling and bicycle racing became immensely popular in the late 19th and early 20th centuries before the advent and rise of motorsports. This is exemplified by the largely popular six-day races that were held in Madison Square Garden, with Marshall “Major” Taylor being one of the most prominent United States sports figures in the early 1900s (1). This popularity is also illustrated in other parts of the world, as seen with the creation of the Tour de France in 1903, which is now one of the most-watched sporting events in the world. This global enthusiasm for cycling has only grown over the years, leading to an estimated one billion bicycles being ridden worldwide (2). These bicycles are often used for recreation, transportation, or a combination of the two. The UN Environment Program notes that in some countries, 60%–90% of transportation is done by bicycle (3). Within the United States, approximately 112 million Americans, or 35% of the population, rode a bike at least one time in 2024. This is the largest percentage of Americans who participated in cycling since PeopleForBikes’ U.S. Bicycling Participation Study began in 2014 (4, 5). Youth participation in recreational cycling has also increased in recent years, with 56% of American youth riding bikes in 2024. The recent increase in cycling in the United States can be partially explained by the COVID-19 pandemic, which propelled interest in utilizing cycling for both transportation and recreation (6, 7). Cultural inequities regarding bicycles and limited access to safe cycling are believed to have kept the sport from becoming mainstream in the United States. Still, BMX cycling participation has grown tremendously over the last decade, rising from 1.86 million in 2012 to 4.46 million participants in 2023 (8). While many factors can be attributed to its growth, the emphasis that USA BMX has on building venues and providing inclusionary programs designed to promote new ridership stands out. BMX is also being promoted as a family-friendly sport, offering everyone opportunities to participate, from young kids to adults (9).

In the sport of mountain bike racing, the National Interscholastic Cycling Association (NICA) has harnessed critical knowledge and processes to create a scalable and robust youth development model. NICA programs introduce young athletes to cycling by partnering with local middle and high schools (10). These programs cultivate a sense of community, providing resources that help young athletes and their parents remain engaged in the sport. NICA's success is illustrated by the steady growth in the Utah league, which expanded from 320 athletes in 2012 to over 7,000 in 2022 (11). In 2024 alone, over 25,000 youth athletes participated in 32 NICA league programs across the United States (12). However, despite the prominence of BMX and NICA programs, most new riders in the United States have difficulty accessing and growing in the sport due to a lack of safe locations to participate and no clear developmental pathways.

## 2 Methods and expert working group process

This Perspectives paper was developed through a series of structured, web-based working group discussions designed to facilitate expert dialogue and consensus building. The Loma Linda University Health institutional review board determined that this work does not meet the definition of human research (IRB# 5260207). Participants were recruited through USA Cycling coaching networks, youth-cycling program listservs, and professional contacts of the authors. This yielded a purposive convenience sample of coaches and program leaders in youth cycling. The final working group and contributor pool included 95 identified individuals, most of whom held USA Cycling coaching licenses. This included 76 males and 19 females. Participants collectively represent extensive experience across the cycling landscape, with many having decades of involvement in athlete development, coaching, and organizational leadership within the United States and internationally. Most contributors are based in the United States, with representation spanning East to West Coast regions, alongside a smaller number of internationally based participants.

Notwithstanding that most members of the working group were affiliated with the National Governing Body for Olympic Sport, participants represented a range of program types from school-based leagues, community clubs, and National Governing Body-affiliated programs. The themes reported here reflect convergence across multiple discussions rather than individual anecdotes. Discussions ran from late 2020 through 2021 and focused on sharing practice-based experiences and identifying common developmental themes rather than collecting systematic research data. The process culminated in a hybrid session at the 2022 USA Cycling Coaching Summit in Colorado Springs, where the emerging framework was refined. These professional discussions did not include identifiable personal or health information and therefore did not constitute human-subjects research requiring institutional review board oversight. All participants were invited to provide feedback and to be acknowledged individually as per publication guidelines. Some individuals chose to be listed as members of the working group, while others declined recognition.

While detailed individual-level demographic data (e.g., city of residence, years in cycling) were not systematically collected, the working group reflects a broad cross-section of the cycling community in terms of roles, disciplines, and regions. Contributors include professional coaches; members of national governing body coaching and administrative staff; academics with expertise in physiology, coaching education, psychology, and related disciplines; and practitioners working across the full developmental continuum, from youth riders who are just learning to ride through to Olympic and professional-level athletes. Represented disciplines include road, track, mountain bike, cyclocross, and emerging off-road formats. In addition, senior administrators from major cycling clubs, representatives of independent coaching organizations, and other stakeholders contributed to the development and refinement of this framework.

The discussions were structured around the American Development Model (ADM) and related Long-Term Athlete Development (LTAD) frameworks as detailed in Table 1. Each call focused on a specific ADM/LTAD stage and used a cloud-based slide deck with key concepts and prompt questions. This covered ADM 1–2 (“Love to Ride”), ADM 2–3 (“Building for the Future”), ADM 4a (“Performance Programs, Participation and Success”), and ADM 4b (“Performance Programs, High Performance”). ADM 5 (“Fit for Life”) was acknowledged as a viable end point but not a primary focus, as the working group concentrated on developmental pathways.

Within each session, we examined ADM-level goals, target populations, scalable program models, desired rider qualities, participant goals, and riding environments, including safety, coaching roles, and progression. Practical factors such as costs, equipment, events, training approaches, and related considerations were also addressed. Each discussion followed a common set of guiding questions. The facilitator (Sean Wilson) captured comments in real time on shared slides, with assistance from a secondary scribe. Later groups iteratively refined statements from earlier calls. Across the series, there were roughly 20 h of facilitated discussion typically delivered in three 1–2-h sessions per ADM level. These ADM-guided discussions served as the organizing framework for synthesis of the concepts outlined in this manuscript.

Some highly detailed elements, such as specific cost structures and full implementation plans, were intentionally excluded from both the discussions and this initial report to maintain focus on core developmental principles. No formal coding, individual interviews, or statistical analyses were performed. However, participants had access to shared documents and could provide written comments directly on the evolving materials. Given the distinctive features of the U.S. sporting context relative to other nations, a systematic comparison of the proposed framework with existing national models was not performed. Albeit, the collective expertise of the group was informed by LTAD and ADM materials from countries such as Canada, several European nations, and Australia. Accordingly, this paper is intended to present a conceptually grounded, cycling-specific LTAD framework for the United States and to highlight key elements for its development, based on an expert-consensus process, rather than to present a formal qualitative analysis or a prescriptive implementation model. The proposed elements are drawn from existing LTAD/Youth Physical Development (YPD) research and practice-based expertise (13–20), with many being evidence-informed pathways, but they have not yet been evaluated as a formal intervention model.

## 3 Cycling landscape and development ecosystem

Although over 50% of American households have bicycles, relatively few people ride bikes regularly. Further, the various cycling disciplines are largely fragmented and disconnected from one another in the United States (21). The lack of cohesion between disciplines and the absence of safe, designated places to ride can make cycling a challenging sport to discover and explore. Cyclists often begin by engaging with a single discipline, and as they develop social connections, they are more likely to continue riding and build the sport into their lifestyle. The specialized nature of bikes and riding venues for each sub-discipline can leave riders secluded in their individual disciplines, limiting their exposure to the richness and diversity of the sport. The ensuing isolation reduces communication between different cycling organizations, which is thought to negatively impact long-term engagement and retention. To counter these effects, cyclists are encouraged to sample various aspects of the sport and build a robust network. By doing this, cyclists will find the elements they enjoy most and will be enabled to develop a healthy appreciation for the sport, processes that increase the potential for long-term participation. The fragmentation and associated barriers to discovery provide context for the LTAD-informed pathways proposed in this manuscript. Figure 1 presents a conceptual socio-ecological model of the US cycling development ecosystem, informed by Bronfenbrenner's ecological systems theory (22, 23). Within this framework, community-based clubs function as LTAD/YPD hubs that interconnect with riders in their local environments and provide support and opportunities for long-term engagement with the sport. The early history of cycling in the United States demonstrates how local clubs, which originated in the late 1800s, played a central role in both the sport and in their communities. These cycling clubs were not just places to learn and grow in the sport, but also served as local community centers (24, 25). The Riverside Bicycle Club, which was established in 1891, is a great example of the culture of cycling that was created in Southern California. Not only did these clubs have clubhouses, many also had velodromes and supported their riders in competitive events (24, 26). One of the most successful clubs in the United States, Century Road Club Association, based in New York City, was founded in 1898. The club continues to be a major driving force for bicycle racing throughout New York City (27). This historical perspective highlights the potential role of community-based programs as hubs for participation and performance. This theme underpins the LTAD-oriented framework proposed here.

## 4 Current youth sport and cycling club models

Popular sports in the United States, such as basketball, football, and baseball, have evolved over the past century as part of the nation's cultural fabric (28). This has led to the creation of youth sports programming through school and community-based club networks, allowing easy entry into these sports. A recent manuscript outlines successful club models for swimming, offering principles that can be applied to other sports (29). Well-developed swim clubs have clear routines, rules, and guidelines to help direct their programs and their long-term development, which reduces complexity and confusion. These rules and guidelines help participants create goals, set staff expectations, and encourage regular performance evaluations. Achieving consistent results and performance at the elite level requires systems that support athletes and their coaching staff as they complete their daily training plans. The impact of well-developed sports programs is also apparent in initiatives like PLAY BALL, which has significantly increased baseball participation through partnerships between Major League Baseball, USA Baseball, and USA Softball (30, 31). PLAY BALL increases participation through the promotion of casual and competitive forms of baseball by creating opportunities for children in the United States and globally. Key to the program's success are the highly coordinated partnerships between local organizers and professional sports organizations, which lower the barriers to entry and provide locally oriented, fun-based activities (30, 31).

Across the United States, there are several successful, youth-oriented, and community-based cycling programs. A prime example is Durango Devo (32), a mountain bike club based in Colorado that has produced numerous athletes who have competed at the Olympic level (33). Even though Durango Devo has developed top-tier performance athletes, their community-centered program has a primary goal of helping kids love to ride. Boulder Junior Cycling (BJC), is a similarly designed youth-oriented cycling program focusing on mountain biking, cyclocross, and road cycling that supports riders interested in recreation and competition (34). BJC works with professional coaches who have developed processes that allow athletes to easily progress from introductory to high-performance levels. Star Track is a track-based program based in New York City, which is a program focused on teaching kids the basics of track cycling (35). Many of the children who participate in Star Track programs are from under-resourced, highly urban areas and would not have had the opportunity to learn about cycling otherwise. Star Track empowers kids, helping them find joy and a sense of accomplishment in cycling. As a testament to the success of their model, Star Track athletes have gone on to become national champions and rise to the highest levels of international sport. These examples demonstrate that when community-based structures, coaching, and venues align, cycling programs can simultaneously foster participation and high performance.

While there are clubs in the United States that have robust and successful models, not all clubs are like this. The infrastructure for community cycling clubs continues to evolve along with society. Smaller performance-oriented competitive teams currently dominate the competitive landscape in the United States over those with a stronger emphasis on community engagement and enjoyment of cycling. As a result of this shift to high performance in competition, many of the most experienced riders and mentors, who used to work with all levels of riders within cycling communities, are now professional coaches working with individual athletes or are team directors. Consequently, many of the local clubs that cater to introductory ridership lack access to top-ranked coaches and mentors, which makes it difficult to establish quality cycling programs. The lack of strong support for new and novice riders has led them to be less connected to the knowledge needed to find their way through the continuum of the sport and build long-term success. The result is that many budding cyclists have poor introductory experiences, fail to become fully engaged, and ultimately leave the sport. The critical gaps in support and continuity are a key rationale for the development of ADM/YPD-aligned pathways and engagement strategies outlined in subsequent sections.

## 5 Pathways for youth cycling engagement and development

When evaluating the appropriate developmental pathways within the sport of cycling, it is important to keep in mind that cycling is much broader than just competition (36). Social engagement in the sport of cycling with one's peers is viewed as key in retaining athletes, both within and outside of the competition sphere. Failure to retain riders is likely because they are not fully engaged. In recent years, gravel events have gained significant traction by catering to athletes who wish to socialize and compete. These events maximize engagement and encourage athletes to develop. Building pathways within all disciplines of cycling that match the interests of younger generations, similar to what gravel is doing currently, can encourage new riders to find the environments they enjoy and engage in the sport.

There are many opportunities to improve the sport of cycling, boost engagement, and improve rider retention. Providing new and varied event formats is one way to increase engagement. This is illustrated by the success of large festival-oriented events including Unbound Gravel (37) and the Sea Otter Classic (38), as well as highly structured, venue-based programs such as those developed by USA BMX (39). Engagement can also be achieved using simple approaches in local environments. Building pathways within local clubs and communities that provide slower paced rides, without the fear of being left behind, can make the sport more social and can appeal to both novice and experienced riders. Regardless of the strategy, the primary goal is to provide fun activities for less advanced riders that promote learning, engagement, and growth. Finding ways to retain cyclists in both competitive and non-competitive communities can not only help to maintain the vitality of the sport of cycling but can also encourage people to use cycling to maintain their physical health. While competition pathways are important, acknowledging that cycling is much broader than just a narrow, competition-oriented segment increases accessibility, making the sport more inviting to new and inexperienced riders (36).

Integrating youth cycling within broader youth sport ecosystems through intentional cross-sport collaboration presents a practical opportunity to enhance holistic athletic development (13, 16, 17). By framing cycling as a transferable training modality that supports aerobic capacity, movement competence, and general athleticism, programs can facilitate constructive “cross-pollination” with multiple sports such as soccer, running, and skiing (15, 40). This approach broadens developmental experiences for youth athletes and creates multiple entry and re-entry points into cycling, thereby strengthening participation pathways and deepening the long-term talent pipeline without sacrificing health or enjoyment (14, 41, 42). Scalable pathways for athlete development in cycling are expected to improve recruitment and retention of riders in the sport. These pathways may involve introducing novice cyclists at an early age through their local schools or parks and recreation programs, while more experienced riders may join cycling clubs, which serve as local community centers. All of these programs would be designed to be safe, accessible, and inclusive. As athletes gain experience, they would be presented with opportunities to graduate into more advanced cycling clubs. When successful, a large proportion of novice cyclists will learn to love the bicycle and become competent, confident, and capable of riding in various environments. Local clubs can provide additional opportunities for riders who are interested in specific disciplines, such as road, track, BMX, or mountain biking. Clubs can provide infrastructure and support for individuals as they engage with multiple aspects of the sport, whether it be for transportation, health and wellness, or competition. Riders interested in competition will have opportunities to join racing programs that offer clearly defined pathways for advancement at the local, regional, national, and international levels. Access to well-designed programs will allow athletes to progress seamlessly through the developmental stages and be challenged in ways that foster growth. As riders advance, they will gain knowledge, expertise, and develop a sense of purpose in the sport. Development-focused programs not only increase a person's enjoyment of riding bicycles but also improve retention and engagement with the sport. When successful, these individuals will incorporate cycling as part of their lifestyle (43). This conceptualization of scalable pathways sets the stage for the subsequent discussion, which links cycling-specific ADM levels to contemporary Youth Development models and to the distinctive governance and funding structures of youth sport in the United States.

## 6 An integrated YPD - LTAD framework for cycling in the United States

The development of scalable pathways for cyclists is crucial as it will impact sport participation through individual athlete growth and retention. Systemic changes that increase participation will expand public awareness, leading to improved access and increased resources and funding for the sport of cycling. As a result, the increased capacity, knowledge, and passion will create a feed-forward effect, with individuals taking on leadership as policy makers, directors, coaches, and mentors, while continuing to participate in the sport. Youth physical development principles provide a foundation for designing pathways that deliberately build strength, movement competence, and confidence so that more riders can participate safely and sustainably over time, ideals that blend with the LTAD framework (16–19, 44, 45). In the YPD model, physical qualities are viewed as trainable throughout childhood and adolescence. Muscular strength and fundamental movement skills provide requisite support for developmentally appropriate, LTAD-aligned progression across the lifespan (13, 16). The YPD elements our analysis focused on are illustrated in Figure 1 through LTAD-YPD hubs. These programs are established to deliver stage-appropriate agility, balance, coordination, and speed building skill and strength using cycling specific experiences within ecological systems frameworks (16, 22, 23, 46).

**Figure 2:**
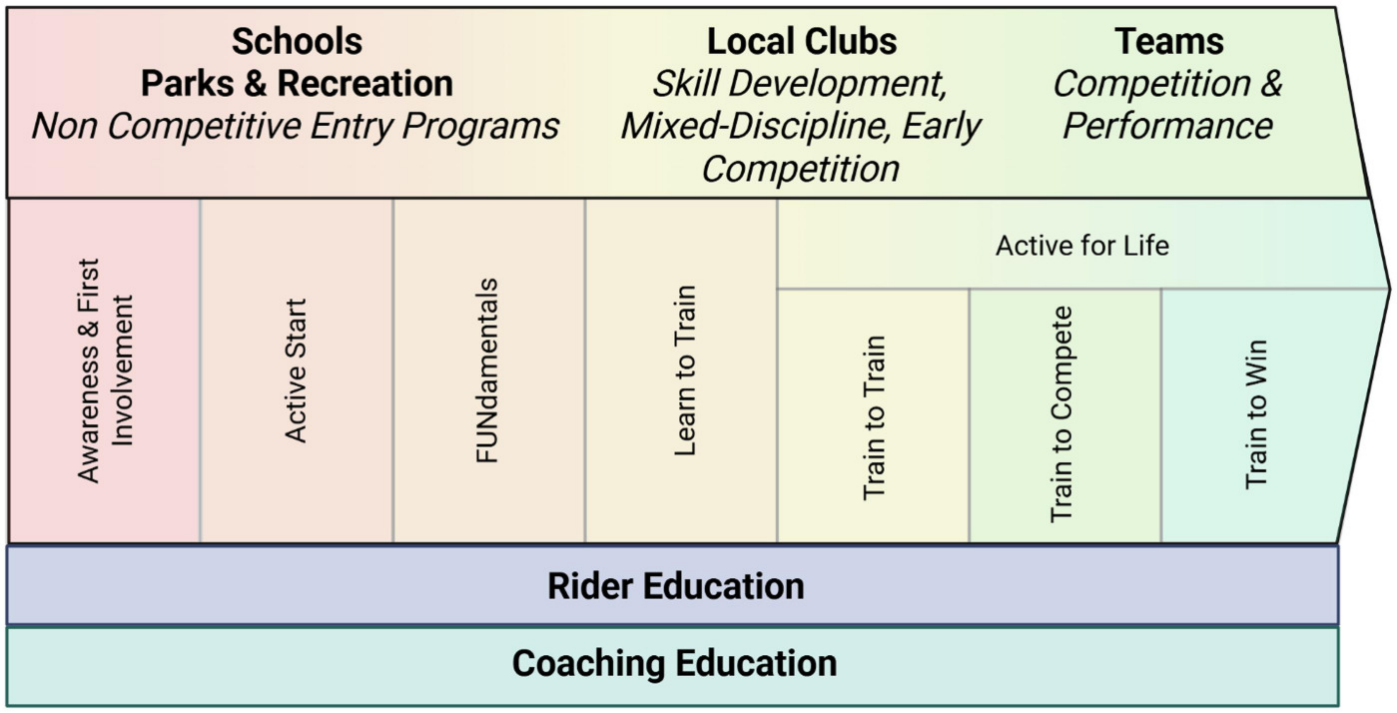
Long-Term athlete development model for cycling in the United States. The figure illustrates the progression of cyclists from novice youth to elite competitors and lifelong participants. Rider development requires strong foundational support from Youth Physical Development principles, grounded in robust coaching and rider education. The model begins with novice youth entering the sport through schools and parks and recreation programs, although riders may enter at any age. This initial focus is on developing a love for cycling through engagement in non-competitive programs. Subsequent developmental stages follow the LTAD framework: Active Start and FUNdamentals in early childhood, emphasizing basic movement and cycling skills and enjoyment; Learn to Train in late childhood to early adolescence, introducing local cycling clubs and initial competitive experiences; Train to Train during adolescence, emphasizing endurance and technical skills; Train to Compete in late adolescence to early adulthood, transitioning to racing teams; and Train to Win in adulthood, focusing on elite competition and performance optimization. The model culminates in the Active for Life stage, which spans adulthood and emphasizes lifelong participation and enjoyment of cycling for transportation, recreation, and health. This progressive pathway supports cyclists at every stage of development, from initial exposure to competitive excellence and sustained engagement in the sport of cycling. Created in BioRender. Wilson (2026) https://BioRender.com/nqzj3jy.

Successful developmental pathways designed for cycling in the United States must not only be best-practice and evidence-based but must also resonate with society (47). Although we do not have any perfect systems in the United States, both USA BMX and NICA, whose programs are grounded in sporting best-practices, are producing positive results. Design elements for a strategically crafted and scalable developmental pathway in the United States can be drawn from other sports as well as from other countries, including Canada, Ireland, Scotland, the Netherlands, Australia, and elsewhere (48–52). Other sports in the United States have implemented pathways strongly associated with LTAD models including USA Hockey, USA Triathlon, USA Swimming along with other National Governing Bodies (53–56). LTAD models provide conceptual frameworks that give key guidance on developmentally appropriate pathways, promoting sport participation across the lifespan (Figure 2). These pathways consider an athlete's emotional, physical, mental, and social growth as they advance in their sport, aiming to foster long-term health and wellness. Complementing the LTAD framework, YPD emphasizes that most physical qualities are trainable throughout childhood and adolescence when sequenced appropriately. Fundamental movement skills and development of muscular strength underpin safe, enjoyable, and inclusive participation (44, 45, 57).

LTAD models are based on progress through multiple developmental stages that allow athletes to grow and enjoy a sport throughout their lifetime. As described in the United States Olympic and Paralympic Committee's American Development Model, Canadian Sport for Life, and other models (14, 20, 53), LTAD outlines a series of hierarchical stages: Awareness and First Involvement, Active Start, FUNdamentals, Learn to Train, Train to Train, Train to Compete, Train to Win, and Active for Life (Figure 2). Within our proposed cycling framework, these LTAD stages provide a structural roadmap, while YPD ideals inform what type of physical development content is emphasized at each stage. For example, broad-based movement skill, basic strength, and play-based cycling experiences are prioritized early in the developmental process with progressive layering of more targeted strength, power, and conditioning work built into training programs as riders mature (17, 18, 44, 45, 57).

Awareness and First Involvement is a precursor to being engaged in sport. The goal is to ensure a positive first experience with physical activity and sports. Next, Active Start aims to help young children under six develop foundational movement skills and connect them together for future athletic endeavors. Activities seek to improve agility, balance, coordination, and basic motor skills. During the FUNdamentals stage, children between the ages of six and nine develop fundamental movement and sports skills through fun activities. Once they enter the Learn to Train phase, children between eight and twelve years old are encouraged to participate in multiple sports to help develop elementary technical skills and continue their growth of foundational sports skills. The Youth Physical Development ideals underscore the value of multi-sport participation at introductory stages of sporting engagement. Varied experiences, including progressive neuromuscular training and diverse cycling activities such as off-road riding, skills courses, and game-based play, support broad physical development and help reduce early specialization pressures (13, 58, 59).

As riders build capacity and skill, youth ages eleven to sixteen will enter the Train to Train phase where they will use structured training approaches to strengthen physical conditioning, refine their sport-specific skills, and develop the ability to focus and set goals. The Train to Compete stage is then geared toward teenage and young adult athletes, ages 15–23, who are focused on optimizing sport performance through intense sport-specific training. Athletes who enter the Train to Win stage are generally over 18 years old and are focused on achieving peak performance at the highest levels of competition, such as the Olympics. These athletes have highly personalized plans for training and competition. Applied YPD principles at these stages include systematically progressing strength and power training, integrating cycling-specific and off-bike conditioning. There is also increased individualization of training load based on an individual's maturation and training history while simultaneously maintaining an emphasis on overall health and well-being (57).

The hope is that mature athletes will enter the Active for Life stage in their post-competitive years regardless of whether they reach the pinnacle of sport or compete at lower levels. The primary goal is for these athletes to have a physically active lifestyle, remaining healthy and engaged in sports and other recreational activities throughout their life. Ensuring appropriate athlete development will promote a healthy lifestyle beyond just performing at a high level in competitions. Long-term engagement is achieved by encouraging athletes, parents, and coaches to avoid excessive amounts of training and early specialization. Avoiding overuse injuries and burnout can also help keep athletes engaged throughout their lives (41). The framework that LTAD provides can guide athletes, parents, coaches, and support systems as they make critical decisions about the direction of an athlete's training and development (40, 46, 60, 61). When integrated with YPD, this framework also guides decisions about when and how to introduce strength, power, and specific conditioning elements, ensuring that developmental pathways in cycling enable athlete performance while simultaneously protecting their health (57).

Considering that the United States lacks a cohesive national development program for cycling, it is impressive that numerous Olympic and world-class cyclists come from the United States. The success of the United States is likely a testament to the country's large population and the affluence of the participants (62). The ability to develop top cyclists is also possible due to crucial pieces of infrastructure that exist in select locations, such as Durango and Boulder, Colorado, which enable athletes with passion, drive, and affluence to excel within those regions. Beyond the scholastic systems that NICA is introducing and which exist at BMX venues, local infrastructure and support is often sporadic and grounded in local interest. Although evidence and best-practice informed systems to support pathways into cycling are expanding, they still only exist in a few regions of the United States (32, 34). Embedding LTAD and YPD ideals within emerging and existing programs offers a way to make physical development pathways more consistent and scalable. Such integration will serve youth in a wider range of communities, who can then access developmentally appropriate cycling opportunities regardless of where they live or their access to resources (63–66).

## 7 Discussion

The present manuscript aims to offer cycling programs in the United States a practical framework for developing long-term pathways in the sport. Our discussions focused on what currently exists in the United States ecosystem at a high level and then considered how to design generalized frameworks that respond to local environments and resource constraints at each developmental level through the lens of the ADM/LTAD model. In this discussion section, we synthesize those working-group themes and highlight how they can inform implementation in diverse local contexts. We recognize that communities differ in resources and cycling opportunities, so local structures must adapt to those realities rather than strive for uniform conditions.

It is notable that the working group identified elements aligned with broader YPD models and their evolution from traditional LTAD frameworks (13, 16). At each ADM level, participants mentioned the importance of safety, fun, and socialization. The resulting framework can be viewed as a cycling-specific, YPD-informed extension of LTAD that prioritizes inclusive participation and lifelong engagement rather than solely talent development. At the introductory ADM levels there was coordinated discussion of how to achieve these outcomes, assessing the importance of developing safe places to ride, play-based activities, and non-competitive pathways in that context (17, 18). This is particularly important if we are to approach physical activity from a holistic perspective that spans across the various lifestyle pillars (13, 16, 67), enabling participants to feel more socially connected with peers, make healthier lifestyle choices, and experience improved mental health and wellness (65, 66). These elements are consistent with bioecological perspectives on development (23). Taken together, these themes support embedding contemporary YPD principles within cycling-specific ADM stages so that riders become competent, confident, and socially connected participants rather than simply progressing through competition tiers. Building on this synthesis of LTAD, YPD, and bioecological principles, further theoretical grounding can be drawn from established youth sport development frameworks.

The framework presented for cycling aligns closely with established youth sport development theory. In particular, the Personal Assets Framework (PAF) conceptualizes how dynamic interactions among activities, relationships, and settings shape positive developmental outcomes in youth sport, including the development of personal assets such as competence, confidence, connection, and character (68–72). These assets, in turn, underpin sustained participation, ongoing personal development, and performance where appropriate. Within the proposed LTAD/YPD-informed cycling model, these elements are represented within the ecosystem structure depicted in Figure 1. These are further elaborated in Tables 1, 2. Developmentally appropriate cycling activities are embedded within the LTAD/YPD pathways centralized in the hub, where “right age, right stage” programming is delivered across disciplines and developmental levels. Relationships, including those among athletes, coaches, peers, and mentors, are represented by the interconnected network surrounding the hub. Coaching systems and community engagement then serve as primary drivers of athlete experience, development, and retention. The settings described in the PAF are reflected in the multi-level ecosystem illustrated in Figure 1, which incorporates school-based programs, community clubs, parks and recreation systems, and national governing bodies to provide multiple entry points and sustained engagement opportunities within a cohesive yet decentralized developmental structure. Consistent with broader positive youth development through sport scholarship and PAF-informed empirical work (69, 73), this model also acknowledges that poorly aligned activities, relationships, or settings may increase risk for dropout and disengagement. This often manifests through reduced enjoyment, perceived competence, or social support, as highlighted in systematic reviews of youth sport attrition (74).

**Table 2:** System-level conditions for implementing LTAD/YPD-informed cycling pathways across essential elements and applied considerations.

Essential elements	Key components (activities, relationships, settings integration)
Physical and emotional safety	Developmentally appropriate riding activities that progressively build skills and confidence Supportive coach–athlete interactions and peer environments (relationships) Safe, accessible riding environments including closed venues, trails, and protected infrastructure (settings)
Coaching and development systems	Age- and stage-appropriate training and skill-building activities National evidence-informed competency based coaching education certification with mentorship and continuous development opportunities (relationships) Delivery through coordinated club, school, and national coaching systems (settings)
Accessibility	Inclusive participation activities appropriate for varying skill levels and entry points Outreach and engagement across diverse communities and populations (relationships) Local, community-based programs aligned with LTAD/YPD hubs and school partnerships with collaboration across local, regional, and national Programs (settings)
Affordability	Low-cost or free entry-level activities and scalable program models with costs proportional to participation level Community and organizational support structures to reduce financial barriers (relationships) Resource-sharing across clubs, schools, and community organizations (settings)
Varied opportunities	Multi-discipline cycling experiences and progressive developmentally appropriate challenges and skill development pathways (activities) Opportunities for social engagement, mentorship, and peer interaction (relationships) Diverse participation environments including clubs, events, schools, and recreational systems aligned with available resources (settings)
Infrastructure and support systems	Appropriate equipment access and structured progression in training and competition (activities) Knowledgeable staff, coaches, and mentors supporting athlete development (relationships) Integrated ecosystem of local programs, regional networks, and national governing bodies (settings)

Table illustrates how essential system-level components of cycling development can be understood through the interaction of activities, relationships, and settings, reinforcing alignment with the Personal Assets Framework and the ecosystem structure presented in Figure 1 and expanded on in Table 1.

These PAF elements are further operationalized across the manuscript through the structures presented in Tables 1, 2. Specifically, Table 1 translates the ecosystem model of Figure 1 into stage-specific programming. The table outlines how developmentally appropriate activities, relationships, and settings are configured across LTAD/YPD levels levels to support participant progression, engagement, and the accrual of personal assets. Table 2 then defines the system-level conditions required to sustain these interactions. This includes relevant coaching systems, accessibility, infrastructure, and safety. The table further sketches how activities, relationships, and settings are supported and scaled within real-world environments. Together, Figure 1, Tables 1, and 2 provide an integrated framework linking conceptual structure, applied programming, and system-level implementation. In practice, these tools can be used by organizations to select or develop age-appropriate cycling activities at each developmental stage. Organizations at all levels can design outreach strategies that facilitate connecting youth with their local hubs (e.g., schools, parks, clubs), and build coaching and support systems that align with LTAD/YPD principles.

Importantly, Figure 1, together with Tables 1, 2, illustrates how the Personal Assets Framework can be operationalized within the unique context of U.S. cycling by demonstrating how activities, relationships, and settings interact across multiple ecological levels. Community-based clubs, acting as LTAD/YPD hubs, anchor participation within high-quality local settings, while the surrounding ecosystem layers extend relationships, resources, and opportunities beyond any single program. This integrated perspective emphasizes that athlete development is not solely driven by progression through competitive pathways, but emerges from the quality, accessibility, and alignment of experiences across these interconnected domains. By embedding PAF principles within the structural model of Figure 1 and aligning them with stage-specific programming (Table 1) and system-level supports (Table 2), the framework translates established youth sport theory into a scalable, systems-level approach suited to the decentralized and resource-variable landscape of cycling in the United States.

Recent policy, practice, and scholarly frameworks collectively underscore the necessity of aligning athlete development with coherent, system-wide approaches to coaching education and development. The 2024 Congressional report *Passing the Torch: Modernizing Olympic, Paralympic, and Grassroots Sports in America* (Recommendation #11) identifies athlete development as a system-dependent outcome shaped by governance, funding, and structural priorities, highlighting persistent misalignment between grassroots participation and elite pathways (75). This systems perspective aligns with long-term athlete development (LTAD) models, which are particularly well suited for sports such as cycling because they conceptualize development as a staged, lifelong process requiring developmentally appropriate experiences across physical, psychological, and social domains (42). However, emerging scholarship suggests the need to extend beyond sport-centric and linear models toward more inclusive and flexible approaches. For example, reframing LTAD as Long-Term Activity Development (LTActD) emphasizes lifelong engagement in physical activity through dynamic, non-linear pathways that account for diverse participation trajectories, re-entry points, and broader health and well-being outcomes (57). Similarly, the *International Coach Developer Framework* and the *International Sport Coaching Framework,* advanced by the International Council for Coaching Excellence, position athlete development as holistic and context-sensitive, requiring coaches to adapt to athlete needs across participation and performance pathways (76, 77). The *National Youth Sports Strategy* further reinforces this perspective by emphasizing physical literacy, sport sampling, and inclusive, developmentally appropriate engagement as foundational to lifelong participation and well-being (78). Collectively, these frameworks support the need for clearly articulated, evidence-informed long-term development models that support both athlete engagement and performance while prioritizing well-being, accessibility, and sustained participation across the life course.

Parallel to this, there is strong consensus that high-quality, evidence-informed coaching education and ongoing coach development are essential mechanisms for achieving athlete development outcomes. The Congressional report positions coaching as a critical yet under-supported workforce requiring coordinated policy-level investment, while ICCE frameworks identify coaching as the primary delivery mechanism through which athlete development occurs (76, 77). Foundational coaching literature further demonstrates that effective coaching integrates professional, interpersonal, and intrapersonal knowledge to enhance athlete competence, confidence, connection, and character (79). The Aspen Institute's Project Play initiative, particularly its call to “Train All Coaches,” provides applied evidence that coach training directly influences athlete experience, retention, and long-term engagement, reinforcing coaching education as a scalable system lever (80). Extending this perspective, LTActD highlights the need for coaching approaches that are adaptable, inclusive, and responsive to individuals across varying stages of engagement, recognizing that participation in sport and physical activity is rarely linear and is shaped by broader social, environmental, and motivational factors (57). Taken together, these sources establish that effective athlete development is contingent upon coaching systems that provide both entry-level preparation and sustained professional learning, ensuring coaches develop not only knowledge but also applied skills aligned with long-term, flexible development models. This integrated approach positions coach education as foundational infrastructure for advancing equitable, developmentally appropriate, and lifelong engagement in sport and physical activity.

For cycling specifically, coaching education and coach developer pathways are requisite, non-negotiable components of an LTAD-aligned system (60, 76–78, 80). Nationally coordinated, evidence-informed coaching standards and accessible training that are harmonized across all cycling disciplines can provide a common language for right age, right stage practice, while still allowing local adaptation. Practically, the community-based LTAD/YPD hubs depicted in Figure 1 depend on prepared coaches who can deliver safe, developmentally appropriate experiences across participation and performance pathways. Well trained coaches are key to linking the policy aspirations of documents such as the National Youth Sports Strategy, Passing the Torch, and Project Play directly to the lived experiences of young riders and their families (75, 78, 80).

A further driving force for developing novel engagement strategies is that Gen Z and Gen Alpha participants appear to respond differently to physical activity than previous generations 81, 82). The working group did not explicitly discuss elements related to generational theory. However, there was consistent emphasis on the importance of sustained enjoyment and autonomy in fostering long-term engagement in these younger riders. This focus has become more pressing given the continued decline in physical activity levels and rising obesity and related conditions (83). Supporting younger generations will require contemporary approaches that move beyond the strictly periodized, curriculum driven, and prescriptive models often associated with LTAD (15, 84). Potential strategies include the use of gamification, Ecological Dynamics and Constraints-Led approaches (58, 85, 86), use of fitness applications (87), content delivery through digital platforms, and concepts adapted from other domains, such as the Dialogue, Access, Risk, and Transparency (DART) model developed for businesses to enhance engagement and co-creation of value (88, 89). These approaches collectively align with ecological and participatory models of behavior change (90, 91). They offer low-infrastructure, scalable tools that can be adapted by resource constrained clubs and programs typical of U.S. cycling.

For this expert opinion manuscript, we consciously decided not to attempt to advance broad policy prescriptions based on idealized LTAD or YPD models from countries with greater public investment and infrastructure for sport (92). Instead, and given the journal's international audience, we briefly situate the context of the United States alongside countries with different sport governance structures to inform future planning and potential policy discussions. We focus this discussion on Europe because of key cultural and socioeconomic similarities (93). In many European countries, youth sports, including cycling, receive public funding within relatively coherent policy frameworks. There is often ministerial-level responsibility for sport and coupling to education, youth, culture, or health portfolios (94). EU-wide programs such as Erasmus + Sport further support grassroots participation, health promotion, inclusion, and capacity building in youth sport organizations (95, 96). This multi-level governance strategy, spanning the European union, national, regional, and municipal authorities, makes public funding a central and expected pillar of club and program sustainability. Fundamentally, the frameworks of these other countries align with bioecological views of development as shaped by interacting processes across micro-, meso-, exo-, and macrosystem levels (22, 23). As a result, European LTAD systems can more readily rely on centralized planning and stable public investment when designing cycling development pathways.

In contrast to countries in the EU and elsewhere worldwide, the United States lacks a federal ministry for sport, a unified national youth sport system, or coordinated public funding architecture (92, 94, 97, 98). Youth sport instead emerges from a decentralized, market-driven mix of school programs, non-profits, and private clubs. These are supplemented by patchwork support from federal initiatives such as the National Youth Sports Strategy, general-purpose grants, corporate, small business, and endemic sponsor support, individual donor investors, along with a highly variable state and local recreation funding structure (13, 97, 98). These funding elements are displayed in Figure 1. The resulting sporting environment is strongly shaped by household resources, corporate and individual sponsorship, and local politics, with public agencies playing a supportive but fragmented role. Within this context, a cycling-specific ADM/LTAD/YPD framework must therefore be modular, locally adaptable, and implementable by organizations with highly variable capacity rather than dependent on centralized mandates.

Cycling remains a niche sport within this distributed sporting environment with relatively low participation rates and budgets compared to mainstream sports (99). Youth sport in the United States is estimated to exceed 40 billion dollars per year, with approximately 25.5 million youth ages 6–17 (54% of all youth) participating in organized sport (100). Major youth cycling organizations collectively serve only a small fraction of the roughly 34 million adolescents ages 11–18 (101) and operate on budgets that are orders of magnitude smaller than those of mainstream sports (8, 12, 102). These figures illustrate that, despite high-profile programs and recent gains, cycling occupies a small share of the broader youth sport economy. Cycling therefore remains vulnerable to fluctuations in sponsorship and household spending. The limited budgets for cycling programs constrain program development and long-term growth. These constraints highlight the need for ADM/YPD-aligned pathways that are simple and efficient to deliver. Programs should intentionally leverage community partnerships and local assets. They must also prioritize retention and inclusivity so that scarce resources translate into durable gains in participation.

## 8 The road ahead

A comprehensive LTAD/YPD model for cycling in the United States has the potential to provide continuous and progressive programs throughout various youth developmental stages. Essential elements for a program designed to improve cycling in the United States are outlined in Table 2. An development approach that incorporates these elements is based on the premise that development is holistic, multifaceted, and occurs over time through “right age, right stage” opportunities. In the United States, the absence of a cohesive pathway for developing cycling-specific skills and knowledge potentially hinders individual progress, undermines long-term engagement, and limits the growth of the sport (47, 99). Empowering new riders to fully engage and embrace cycling as a lifestyle will require having well informed and trained coaches who can revitalize and modernize local cycling clubs using LTAD and YPD frameworks and models. The emphasis here is not on building racing team structures, but rather on developing programs that provide opportunities for the discovery and exploration of cycling grounded in best-practice and evidence-based approaches.

## 9 Realizing the vision

Within this proposed LTAD-aligned roadmap for U.S. cycling, the outcomes from developing evidence-based cycling communities founded on LTAD/YPD principles are exciting and significant. These communities have the potential to increase the number of domestic cyclists participating in the sport. They will help newcomers become confident and competent riders, support those who use cycling for transportation, and provide opportunities to cyclists who use bicycles for fitness, recreation, and fun. Building local infrastructure and support, and improving developmental pathways across the continuum of the sport, is anticipated to increase ridership and deepen the level of competition. Participants who progress through these pathways are likely to return as coaches, organizers, and policy advocates, which will further strengthen cycling communities. Public interest and traction for the sport are expected to increase, leading to cycling becoming more fully integrated into American culture and, for many, a sustainable lifestyle.

## Data Availability

The original contributions presented in the study are included in the article/Supplementary Material, further inquiries can be directed to the corresponding author.
